# Diagnostic value of FDG PET/CT imaging in patients with surgically managed infective endocarditis: results of a retrospective analysis at a tertiary center

**DOI:** 10.1007/s12350-020-02457-x

**Published:** 2020-12-22

**Authors:** Sabine Julia Maria Sag, Karin Menhart, Jirka Grosse, Florian Hitzenbichler, Frank Hanses, Arno Mohr, Bernd Salzberger, Matthäus Zerdzitzki, Michael Hilker, Leopold Rupprecht, Dirk Hellwig, Christof Schmid, Lars Siegfried Maier, Can Martin Sag

**Affiliations:** 1 Department of Internal Medicine II, University Hospital Regensburg, Franz-Josef-Strauß-Allee 11, 93053 Regensburg, Germany; 2 Department of Nuclear Medicine, University Hospital Regensburg, Regensburg, Germany; 3 Department of Infection Prevention and Infectious Diseases, University Hospital Regensburg, Regensburg, Germany; 4 Department of Cardiothoracic Surgery, University Hospital Regensburg, Regensburg, Germany

**Keywords:** Inflammation, PET, image interpretation

## Abstract

**Background:**

We assessed the diagnostic value of FDG PET/CT in a real-world cohort of patients with surgically managed infective endocarditis (IE).

**Methods:**

We performed a retrospective analysis of all patients hospitalized in a tertiary IE referral medical center from January 2014 to October 2018 fulfilling the following criteria: ICD-10 code for IE and OPS code for both, heart surgery and FDG PET/CT.

**Results:**

Final analysis included 29 patients, whereof 28 patients had surgically proven IE. FDG PET/CT scan was true-positive in 15 patients (sensitivity (SEN) 56%) and false-negative in 12 patients. Combination of Duke criteria (DC) with FDG PET/CT scan resulted in gain of SEN for all patients with confirmed IE (SEN of DC 79% vs SEN of combination DC and FDG PET/CT 89%), driven by a relevant gain in PVE patients only (SEN of DC 78% vs SEN of combination DC and FDG PET/CT 94%). Interestingly, higher prosthesis age was observed in patients with false-negative scans.

**Conclusions:**

We found a SEN of 56% for FDG PET/CT in a real-world cohort of patients with surgically proven IE which was associated with a 16% gain of IE diagnosis in patients with PVE when combined with DC.

**Supplementary Information:**

The online version of this article (10.1007/s12350-020-02457-x) contains supplementary material, which is available to authorized users.

## Introduction

Despite medical advancements management of infective endocarditis (IE) is still challenging both from a diagnostic as well as from a therapeutic point of view. Particularly, diagnostic work up in case of suspected prosthesis valve endocarditis (PVE) can be error-prone.^1^,^2^ Therefore, 2015 ESC IE guidelines incorporated further imaging modalities, including cardiac FDG PET/CT for detection of abnormal periprosthetic inflammation activity, to improve diagnostic accuracy of Duke criteria (DC).^3^ Notably, ESC guidelines are based on studies in which IE diagnosis in PVE and cardiac device-related infective endocarditis (CDRIE) patients was predominantly made by expert teams, respectively, by Duke classification.^4^-^6^ Likewise, more recent studies investigating the diagnostic value of FDG PET/CT in suspected IE have mainly used expert opinion or DC as reference standard for definite IE diagnosis.^7^-^10^ However, an assessment of the diagnostic value of FDG PET/CT via surgical confirmation as reference standard for definite IE is largely missing. This lack of definite surgical diagnosis may complicate interpretation of FDG PET/CT sensitivity (SEN) and specificity, which could have contributed to the high variation of FDG PET/CT SEN in the context of IE diagnostics.^4^-^10^

The aim of this retrospective analysis was to evaluate the diagnostic value of preoperatively performed PET imaging during a 4 years observation period in a real-world cohort of patients undergoing heart surgery due to suspected IE at a tertiary IE referral center. Furthermore, we wanted to investigate the potential gain of SEN by including PET results into traditional Duke classification for identifying IE patients. In addition, we aimed to investigate confounders that resulted in false-negative or false-positive FDG PET/CT results.

## Methods

### Patient cohort

We retrospectively reviewed medical records of all patients hospitalized in a tertiary IE referral medical center from January 2014 to October 2018 fulfilling the following criteria: International Classification of Diseases (ICD)-10 code for IE and code for both, heart surgery and FDG PET/CT according to the German classification of operations and procedures (OPS code). Only patients with preoperatively performed FDG PET/CT and surgically proven diagnosis were included. The institutional Ethics committee approved this study and waived the necessity to obtain informed consent.

Demographic, microbiological and echocardiographic data of all included patients were gathered.

### FDG PET/CT imaging and image interpretation

In 17 patients a Biograph 16 PET/CT scanner (CTI-Siemens, Erlangen, Germany) consisting of a 16-slice multidetector CT (.5 s per revolution) was used; in 12 patients a Biograph mCT 40 FLOW PET/CT scanner (CTI-Siemens, Erlangen, Germany), consisting of a 40-slice multidetector CT (.5 s per revolution) was used.

After a fasting period of at least 6 hours, 3 MBq ^18^F-FDG per kilogram body weight were injected intravenously (254 ± 43 MBq). Please note that no specific dietary requirements such as a low-carb/ high-fat diet were recommended to patients. The patients’ blood glucose level was strictly controlled to be below 150 mg/dL (8.32 mmol/L). To increase renal tracer elimination, patients received an injection of 20 mg furosemide as well as intravenous hydration shortly after ^18^F-FDG injection.

To minimize muscular ^18^F-FDG uptake, patients were advised to stay in a quiet lying position. Warming blankets were used to avoid freezing of the patients and to keep potential tracer accumulation in brown fat tissue to a minimum. Patients were instructed to void the bladder prior to scanning and to remove all metal parts.

After a waiting period of about 60 minute post-injection, the PET/CT acquisition was performed. Using the Biograph 16 PET/CT scanner, images of the trunk were acquired with elevated arms (pelvis to skull or skull base). Depending on the patient size and clinical indication, six to eight overlapping bed positions with 3 minutes of PET acquisition time each were used. Using the Biograph mCT 40 FLOW PET/CT scanner, images of the whole body (skull to feet) were acquired using the continuous bed move (torso: 0.8 cm/min, legs: 1.1 cm/min). The same area was covered by a low-dose CT scan (tube current 50 mAs, tube voltage 120 kV). No contrast agents were given.

PET images (slice thickness 5 mm) were corrected for random coincidences, decay, scatter, and attenuation and reconstructed iteratively using the ordered subsets expectation maximization algorithm (OSEM) with four iterations and eight subsets. PET images were scaled to allow SUV measurements. PET and CT images were checked for breathing artifacts. Only PET images without ECG gating were used for re-analysis.

FDG PET/CT images were reanalyzed by two independent nuclear medicine physicians blinded to patients´ characteristics, using syngo.via software (version V30, Siemens Healthcare, Germany). Scans with abnormal focal or diffuse ^18^F-FDG uptake (without using a fixed threshold), compared to surrounding blood pool, corresponding to cardiac valve, prostheses or intracardiac devices were considered positive for IE. Attenuation corrected as well as uncorrected images were analyzed separately.

### Modified Duke classification

DC were assessed at the time of admission, at the time of FDG PET/CT and at the end of hospital stay according to 2015 ESC IE guidelines.^3^

### Definition of final IE diagnosis

Final IE diagnosis was made based on intraoperative findings being consistent with signs of acute or subacute infection (such as vegetations or abscesses). Microbiological tissue samples were documented positive when pathogens were successfully cultured or identified by PCR. Histopathological confirmation was gathered when histology was consistent with IE and/or pathogens could be identified.

### Statistical analysis

Data were analyzed using the SPSS statistical software package (SPSS 23.0, IBM SPSS Statistics, Armonk, New York, USA). Descriptive statistics are presented as median and interquartile range (IQR) for continuous data and as number and percentages for categorical data. Fisher´s exact test was used to compare median values for independent data. Categorical parameters were evaluated by Chi-squared test.

SEN was calculated with the following formula: number of true-positive test results/number of patients with surgically proven IE.

Due to selection of our patient cohort with high IE probability determination of specificity as well as positive and negative predictive value was not viable.

## Results

**Figure 1:**
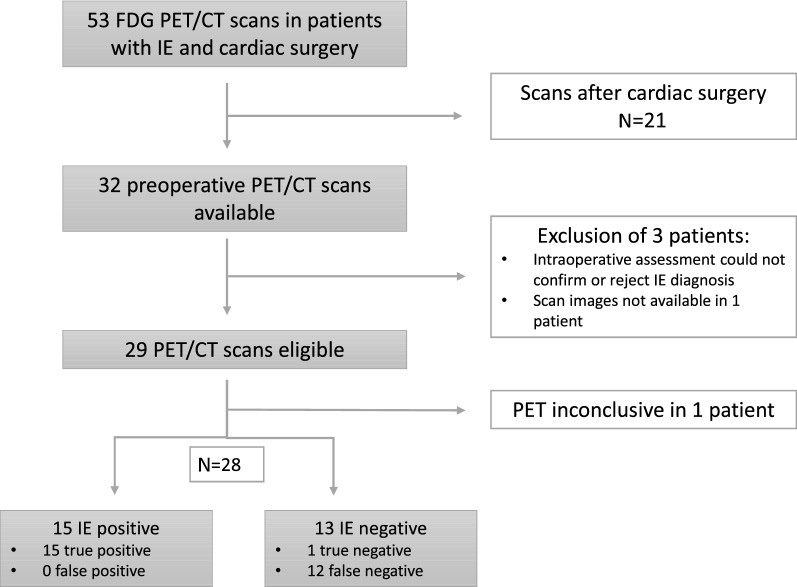
Flowchart of retrospective FDG PET/CT selection in patients with definite IE before cardiac surgery

Between January 2014 and October 2018, 53 FDG PET/CT scans were performed in patients with both, ICD code for IE and OPS code for heart surgery. Out of 53 screened patients, N = 21 patients were excluded because FDG PET/CT scan was performed post-surgery. In two patients, intraoperative assessment could not confirm or reject IE diagnosis. In one patient, no FDG PET/CT images were available for re-analysis. Hence, 29 FDG PET/CT scans were eligible for retrospective analysis (Figure 1).

### Clinical characteristics

Clinical characteristics of all 29 patients are displayed in Table 1. Median age was 64 years (IQR: 58 to 69 years) and the majority of patients was male (86%). 8 patients (28%) died during hospital stay. 69% of patients were referred from other hospitals. 7 patients (24%) had native valve IE (NVE), 18 patients (62%) had PVE and 4 patients (14%) had isolated cardiac device-related IE (CDRIE). Vegetations were identified in 23 patients (79%) by echocardiography and main IE affection site was aortic valve region. Median vegetation size was 17 mm (IQR: 12 to 23 mm] and abscess formation was detected echocardiographically in 4 patients. 50% of PVE patients had bioprostheses and median time since prosthesis implantation was 4.0 years (IQR: .8 to 9.3 years). CRP at the time of admission was 148.7 mg/dL (IQR: 66.7 to 281.3 mg/dL) and blood cultures were available in all patients. 26 patients (90%) had positive blood cultures and the most frequent causative pathogen was *Staphylococcus aureus* (58%). In 28 out of all 29 patients IE was proven surgically (1 patient had no in situ IE). Microbiology was available in 24 patients (83%) and histopathology in 13 patients (45%). FDG PET/CT was performed at a median time of 10 days (IQR: 7 to 20 days) in case of referral from another hospital and at a median time of 4 days (IQR: 3 to 8 days) after admission to our tertiary center. Time between first positive blood culture and FDG PET/CT was 8 days (IQR: 5 to 15 days). CRP at the time of FDG PET/CT was 87.3 mg/dL (IQR: 39.0-117.3 mg/dL) and 26 patients (90%) received antibiotic therapy at the time of the scan.

**Table 1:** Clinical characteristics in patients with surgically managed IE and preoperatively performed FDG PET/CT

	N = 29
Demographics	
Age, median [IQR], y	64 [58–69]
Sex (male), n (%)	25 (86)
Diabetes mellitus, n (%)	4 (14)
Prior history of IE, n (%)	3 (10)
Intrahospital mortality, n (%)	8 (28)
Intrahospital IE, n (%)	3 (10)
Prior admission to other hospital, n (%)	20 (69)
Echocardiographic data	
Native valve, n (%)	7 (24)
Impairment of LVEF, n (%)	8 (29)
Time to initial TOE, median [IQR], days	0 [−3 to 2]
Time to TOE at tertiary hospital, median [IQR], days	2.5 [1–4]
Initial TOE-negative, n (%)	1 (4)
Primary Duke criterion-positive, n (%)	27 (93)
Vegetation, n (%)	23 (79)
Vegetations only	15/23
IE affection site^a^, n (%)	
Aortic	16 (55)
Mitral	11 (38)
Pulmonary	1 (3)
Tricuspid	3 (10)
Cardiac device	7 (24)
Vegetation size, all, median [IQR], mm	17 [12–23]
NVE, median [IQR], mm	15 [12–25]
PVE, median [IQR], mm	16 [11–21]
Abscess, n (%)	4 (14)
Fistula, n (%)	0 (0)
Prosthetic valve dehiscence, n (%)	1 (6)
Paravalvular leakage, n (%)	3 (17)
Prosthetic valve IE, n (%)	18 (62)
Mechanical, n	4/18
Biological, n	9/18
Reconstruction, n	5/18
Including replacement of ascending aorta, n	3/18
Time since implantation, median [IQR], y	4.0 [0.8–9.3]
Valves implanted >1 year, n	13/18
Valves implanted <3 months, n	4/18
Valves implanted 3–12 months, n	1/18
Cardiac device IE, n (%)	7 (24)
Isolated device infection, n	4/7
Pacemaker, n	3/7
ICD, n	2/7
CRT-D, n	2/7
CRT-P, n	0/7
Time since implantation, median [IQR], y	2 [1–4]
Device implanted >1 year, n	6/7
Device implanted <3 months, n	1/7
Device implanted 3–12 months, n	0/7
Microbiology	
CRP at the time of admission, median [IQR], mg/L	148.7 [66.7–281.3]
Leukocytes at the time of admission, median [IQR], /nL	11.8 [8.9–15.3]
PCT at the time of admission, median [IQR], ng/mL	1.33 [1.02–19.52]
Blood cultures available, n (%)	29 (100)
Blood cultures positive, n (%)	26 (90)
Primary Duke criterion positive, n (%)	22 (76)
Causative pathogen, n	
*Staphylococcus aureus*	15/26
Enterococci	1/26
Coagulase-negative staphylococci	4/26
Streptococci	4/26
HACEK	1/26
*Candida* sp.	1/26
Antibiotic therapy before blood cultures, n (%)	6 (21)
Empiric antibiotic therapy, n (%)	25 (86)
FDG PET/CT	
Time to FDG PET/CT since external admission, median [IQR], days	10 [7–20]
Time to FDG PET/CT since admission at tertiary center, median [IQR], days	4 [3–8]
Time between FDG PET/CT and surgery, median [IQR], days	9 [4–16]
Indication for FDG PET/CT	
Inconclusive echocardiography	5 (17)
Other foci/septic emboli	11 (38)
Combination of both	13 (45)
Time from first positive blood culture to FDG PET/CT, median [IQR], days	8 [5–15]
Duration of antibiotic therapy before FDG PET/CT, median [IQR], days	3 [0–6]
CRP at the time of FDG PET/CT, median [IQR], mg/L	87.3 [39.0–117.3]
Leukocytes at the time of FDG PET/CT, median [IQR], /nL	10.7 [7.5–12.8]
Fasting glucose, median [IQR], mg/dL	101 [89–155]
Fever at the time of FDG PET/CT, n (%)	3 (18)
FDG PET/CT during antibiotic therapy, n (%)	26 (90)
Pathogen-directed therapy, n (%)	21 (72)
Vegetation size at the time of FDG PET/CT, median [IQR], mm	11 [5–19]
Inadequate myocardial suppression, n (%)	9 (31)
FDG PET/CT result inconclusive, n (%)	1 (3)
FDG PET/CT positive, n	15/28
True-positive, n	15/15
False-positive, n	0/15
FDG PET/CT negative, n	13/28
True-negative, n	1/13
False-negative, n	12/13
SUVmax in positive FDG PET/CT, median [IQR]	5.8 [4.9–7.6]
Septic emboli detected by FDG PET/CT, n (%)	4 (14)
Modified Duke classification at admission, n (%)	28 (97)
Definite IE, n	16/28
Possible IE, n	7/28
Rejected, n	5/28
Modified Duke classification at the time of FDG PET/CT, n (%)	29 (100)
Definite IE, n	22/29
Possible IE, n	6/29
Rejected, n	1/29
Modified Duke classification at the end of hospital stay, n (%)	29 (100)
Definite IE, n	23/29
Possible IE, n	6/29
Rejected, n	0/29
Definite IE (microbiological, histopathological or surgical confirmation)	
Microbiology available, n (%)	24 (83)
Microbiological confirmation of IE, n	12/24
Culture positive, n	4/12
PCR positive, n	11/12
Histopathology available, n (%)	13 (45)
Histopathological confirmation of IE, n	10/13
Detection of germs, n	6/10
Surgical confirmation of IE, n (%)	28 (97)
Intraoperative abscess, n	11/28

*CRP*, C-reactive protein; *CRT-D*, cardiac resynchronization therapy defibrillator; *CRT-P*, cardiac resynchronization therapy pacemaker; *CT*, computed tomography; *FDG*, ^18^F-fluorodeoxyglucose; *ICD*, implantable cardioverter defibrillator; *IE*, infective endocarditis; *LVEF*, left ventricular ejection fraction; *PCT*, procalcitonin; *PET*, positron emission tomography; *SUVmax*, maximal standardized uptake value; *TOE*, transesophageal echocardiography

Values represent the median [interquartile range] or numbers (percentages)

^a^The number of IE affection sites is higher than the number of patients because some patients had more than one affection site

### Test results of FDG PET/CT in patients with surgically managed IE

**Figure 2:**
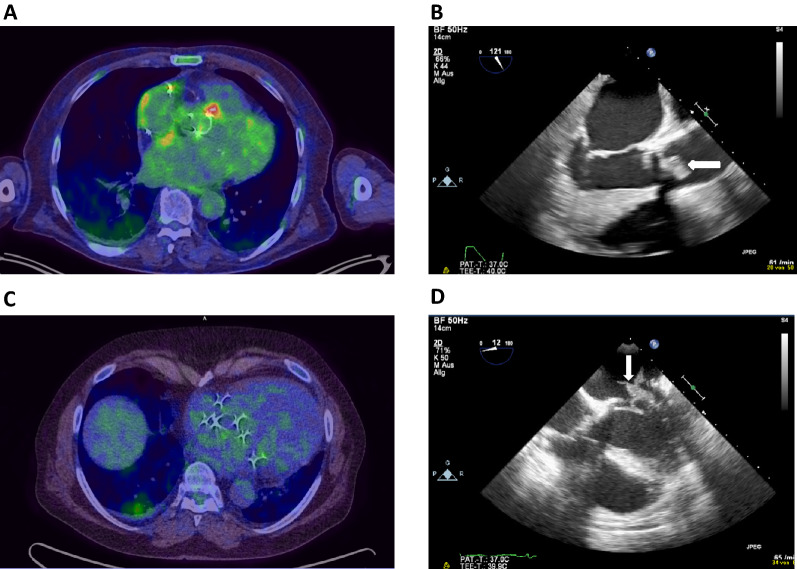
Exemplary FDG PET/CT scans and echocardiographic images illustrating the according endocarditic lesion are presented in this figure for a patient with a true-positive test result (**A** and **B**) and for a patient with a false-negative test result (**C** and **D**)

Out of 29 FDG PET/CT scans only one was inconclusive. Hence, 28 FDG PET/CT scans results were used for further analysis. 15 patients with surgically confirmed IE had a positive FDG PET/CT (sensitivity (SEN) 56%). FDG PET/CT was negative in 13 patients, yet only 1 true-negative. Hence, 12 of 13 negative scan results were false-negative (Table 2). Exemplary FDG PET/CT scans and echocardiographic images illustrating the according endocarditic lesion are presented in Figure 2 for a patient with a true-positive test result (Figure 2A and B) and for a patient with a false-negative test result (Figure 2C and D).

**Table 2:** Two-by-two contingency table for the diagnosis of infective endocarditis via FDG PET/CT

	Reference standard^a^		
	+	−	Total
PET			
+	15	0	15
−	12	1	13
Total	27	1	28

*PET*, positron emission tomography

^a^Reference standard corresponds to intraoperative confirmation of infective endocarditis

**Figure 3:**
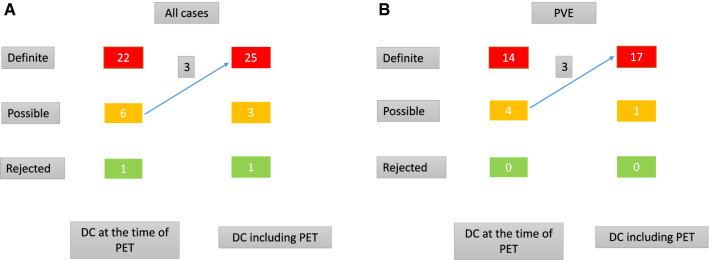
Reclassification of subjects with possible IE according to DC at the time of PET using positive FDG PET/CT result as major DC (**A**: all cases, **B**: PVE)

Out of 15 patients with a positive FDG PET/CT scan, 12 patients had definite IE according to DC. In patients with a negative FDG PET/CT scan, diagnosis of IE according to DC would have been rejected in one patient with surgically confirmed IE. 22 out of 29 patients (79%) fulfilled traditional DC for definite IE at the time of FDG PET/CT. Of these 22 patients, indications for FDG PET/CT were as follows: inconclusive results from echocardiography (N = 4), other foci/septic emboli (N = 7), combination of both (N = 11). By including the FDG PET/CT result as a major DC into Duke classification, 3 further patients could be reclassified as definite IE (Figure 3). Combination of DC with FDG PET/CT scan resulted in a gain of SEN for all patients with confirmed IE (SEN of DC 79% vs SEN of combination DC and FDG PET/CT 89%), driven by a relevant gain in PVE patients only (SEN of DC 78% vs SEN of combination DC and FDG PET/CT 94%). In NVE, SEN of DC did not improve when combined with FDG PET/CT (SEN of DC 83% vs SEN of combination DC and FDG PET/CT 83%). SEN values for DC, FDG PET/CT and combination of both are presented in Table 3. Interestingly, 4 patients had septic emboli that were detected by FDG PET/CT. However, adding this finding as a minor DC did not result in a gain of IE SEN since all of the patients were diagnosed with definite IE according to DC at the time of FDG PET/CT already.

**Table 3:** Sensitivity values of DC, FDG PET/CT, and combination of DC and FDG PET/CT for all patients as well as in NVE, PVE and CDRIE

	SEN DC^a^	SEN PET/CT	SEN DC^a^ + PET/CT
All (N = 28)	79% (60–90%)	56% (37–72%)	89% (73–96%)
NVE (N = 6)	83% (44–97%)	33% (10–70%)	83% (44–97%)
PVE (N = 18)	78% (55–91%)	61% (39–80%)	94% (74–99%)
CDRIE (N = 4)	75% (30–95%)	50% (15–85%)	75% (30–95%)

*CDRIE*, cardiac device-related infective endocarditis; *CT*, computed tomography; *DC*, Duke criteria; *FDG*, ^18^F-fluorodeoxyglucose; *NVE*, native valve endocarditis; *PET*, positron emission tomography; *PVE*, prosthetic valve endocarditis; *SEN*, sensitivity

Values represent percentages (95% confidence interval)

^a^DC at the time of PET/CT

Table 4 depicts clinical characteristics of patients with true-positive and false-negative FDG PET/CT scans. Regarding demographic and echocardiographic parameters no significant differences between groups could be detected, yet a trend toward a greater proportion of female sex (*P* = .053) and more echocardiographic evidence of abscess formation (*P* = .053) could be seen in patients with true-positive scan results. Microbiological, histopathological and surgical parameters did not differ between groups. With respect to FDG PET/CT-specific parameters, true-positive scans showed as expected higher median SUVmax (5.8 [4.9-7.6] vs 3.9 [3.3-4.8], *P* = .006) compared to false-negative FDG PET/CTs. The proportion of patients with inadequate myocardial suppression was not significantly different between both groups.

**Table 4:** Clinical characteristics in IE patients with true-positive vs false-negative FDG PET/CT results

	FDG PET/CT true-positive (N = 15)	FDG PET/CT false-negative (N = 12)	*P* value
Demographics			
Age, median [IQR], y	66 [53–75]	64 [62–67]	.441
Sex (male), n (%)	11 (73)	12 (100)	.053
Diabetes mellitus, n (%)	3 (20)	0 (0)	.1
Prior history of IE, n (%)	1 (7)	1 (8)	.509
Intrahospital mortality, n (%)	5 (33)	3 (25)	.637
Intrahospital IE, n (%)	1 (7)	2 (17)	.411
Prior admission to other hospital, n (%)	12 (80)	12 (80)	.432
Echocardiographic data			
Native valve, n (%)	2 (15)	4 (40)	.198
Impairment of LVEF, n (%)	4 (29)	4 (33)	.793
Time to initial TOE, median [IQR], days	0 [–4–2]	1 [−2–5]	.685
Time to TOE at tertiary hospital, median [IQR], days	3 [1–3.5]	4 [1–6]	.187
Initial TOE-negative, n (%)	1 (7)	0 (0)	.327
Primary Duke criterion-positive, n (%)	14 (93)	11 (92)	.358
Vegetation, n (%)	12 (80)	10 (83)	.651
Vegetations only	8/12	7/10	.951
IE affection site^a^			
Aortic	9 (60)	6 (50)	.603
Mitral	4 (27)	6 (50)	.212
Pulmonary	0 (0)	1 (8)	.255
Tricuspid	3 (20)	0 (0)	.1
Cardiac device	3 (20)	4 (33)	.432
Vegetation size, median [IQR], mm	19 [14–23]	17 [11–22]	1
Vegetation size in NVE, median, mm	20^b^	18^b^	1.0
Abscess, n (%)	4 (27)	0 (0)	.053
Fistula, n (%)	0 (0)	0 (0)	1
Prosthetic valve dehiscence, n (%)	0 (0)	1 (17)	.171
Paravalvular leakage, n (%)	2 (18)	1 (17)	.829
Prosthetic valve IE, n (%)	11 (73)	6 (50)	.212
Mechanical, n	2/11	2/6	.482
Biological, n	6/11	3/6	.858
Reconstruction, n	3/11	1/6	.622
Including replacement of ascending aorta, n	2/11	0/6	.266
Vegetation size, median [IQR], mm	16 [12–21]	14 [11–21]	.825
Time since implantation, median [IQR], y	1.0 [.0–4.0]	6.5 [4.8–14.8]	.035
Valves implanted >1 year, n	6/11	6/6	.049
Valves implanted <3 months, n	4/11	0/6	.091
Valves implanted 3–12 months, n	1/11	0/6	.446
Cardiac device IE, n (%)	3 (10)	4 (33)	.432
Isolated device infection, n	2/3	2/4	.659
Pacemaker, n	2/3	1/4	.27
ICD, n	1/3	1/4	.809
CRT-D, n	0/3	2/4	.147
CRT-P, n	0/3	0/4	1
Time since implantation, median [IQR], y	2.0^b^	2.5 [1.3–3.8]	1
Device implanted >1 year, n	2/3	4/4	.212
Device implanted <3 months, n	1/3	0/4	.212
Device implanted 3–12 months, n	0/3	0/4	1
Microbiology			
CRP at the time of admission, median [IQR], mg/L	168.1 [109.0–278]	103.5 [41.4–343.8]	.637
Leukocytes at the time of admission, median [IQR], /nL	12.6 [9.6–19.1]	11.1 [6.4–14.7]	.704
PCT at the time of admission, median [IQR], ng/mL	1.37 [1.09–23.51]	9.20 [0.86–21.38]	1
Blood cultures available, n (%)	15 (100)	12 (100)	1
Blood cultures-positive, n (%)	15 (100)	10 (83)	.1
Primary Duke criterion-positive, n (%)	13 (87)	9 (75)	.438
Causative pathogen			
*Staphylococcus aureus*	9/15	6/10	1
Enterococci	0/15	1/10	.211
Coagulase-negative staphylococci	3/15	1/10	.504
Streptococci	1/15	2/10	.315
HACEK	1/15	0/10	.405
*Candida* sp.	1/15	0/10	.405
Antibiotic therapy before blood cultures, n (%)	3 (20)	2 (17)	.923
Empiric antibiotic therapy, n (%)	14 (93)	10 (83)	.283
FDG PET/CT			
Time to FDG PET/CT since external admission, median [IQR], days	10 [5–19]	11 [7–30]	1
Time to FDG PET/CT since admission at tertiary center, median [IQR], days	4 [3–7]	5 [3–8]	.449
Time between FDG PET/CT and surgery, median [IQR], days	11 [5–20]	6 [4–12]	.126
Time from first-positive blood culture to FDG PET/CT, median [IQR], days	8 [4–17]	8 [7–15]	.669
Duration of antibiotic therapy before FDG PET/CT, median [IQR], days	1 [0–11]	3 [−6−5]	.605
CRP at the time of FDG PET/CT, median [IQR], mg/L	81.2 [34.4–102.5]	106.0 [71.2–193.5]	.68
Leukocytes at the time of FDG PET/CT, median [IQR], /nL	10.8 [9.2–12.9]	9.4 [6.4–12.9]	1
Fasting glucose, median [IQR], mg/dL	95 [88–138]	103 [93–144]	.697
Fever at the time of FDG PET/CT, n (%)	1 (7)	2 (17)	.522
FDG PET/CT during antibiotic therapy, n (%)	14 (93)	10 (83)	.185
Pathogen-directed therapy, n (%)	11 (73)	8 (67)	.702
Vegetation size at the time of FDG PET/CT, median [IQR], mm	10 [0–16]	17 [9–21]	.4
Inadequate myocardial suppression, n (%)	4 (27)	4 (33)	.706
SUVmax in positive FDG PET/CT, median [IQR]	5.8 [4.9–7.6]	3.9 [3.3–4.8]	.006
Septic emboli detected by FDG PET/CT, n (%)	2 (13)	2 (17)	.809
Modified Duke classification at admission, n (%)	14 (93)	12 (100)	.362
Definite IE, n	9/14	6/12	.462
Possible IE, n	4/14	3/12	.838
Rejected, n	1/14	3/12	.208
Modified Duke classification at time of FDG PET/CT, n (%)	15 (100)	12 (100)	1
Definite IE, n	12/15	9/12	.756
Possible IE, n	3/15	2/12	.825
Rejected, n	0/15	1/12	.255
Modified Duke classification at end of hospital stay, n (%)	15 (100)	12 (100)	1
Definite IE, n	14/15	8/12	.076
Possible IE, n	1/15	4/12	.076
Rejected, n	0/15	0/12	1
Definite IE (microbiological, histopathological or surgical confirmation)			
Microbiology available, n (%)	14 (93)	10 (83)	.411
Microbiological confirmation of IE, n	7/14	5/10	1
Culture-positive, n	1/7	3/5	.098
PCR-positive, n	7/7	4/5	.217
Histopathology available, n (%)	8 (53)	4 (33)	.299
Histopathological confirmation of IE, n	7/8	2/4	.253
Detection of germs, n	4/7	1/2	.858
Surgical confirmation of IE, n (%)	15 (100)	12 (100)	1
Intraoperative abscess, n	7/15	3/12	.247

CRP, C-reactive protein; CRT-D, cardiac resynchronization therapy defibrillator; CRT-P, cardiac resynchronization therapy pacemaker; CT, computed tomography; FDG, ^18^F-fluorodeoxyglucose; ICD, implantable cardioverter defibrillator; IE, infective endocarditis; LVEF, left ventricular ejection fraction; PCT, procalcitonin; PET, positron emission tomography; SUVmax, maximal standardized uptake value; TOE, transesophageal echocardiography

Values represent the median [interquartile range] or numbers (percentages)

^a^The number of IE affection sites is higher than the number of patients because some patients had more than one affection site

^b^Interquartile range could not be calculated as n < 4

In case of PVE type of prosthesis was not different between groups, however both, time since prosthesis implantation (1.0 year vs 6.5 years, *P* = .035) and proportion of patients with prosthesis implanted longer than one year ago (55% vs 100%, *P* = .049), were greater in patients with false-negative FDG PET/CT scans.

## Discussion

In our retrospective analysis of surgically managed IE cases diagnosis of definite IE was confirmed in 28 of 29 patients via intraoperative assessment. This way of definite IE diagnosis is a major strength of our study, because final IE diagnosis was not based on DC or expert opinion only, but was surgically proven, therefore correlation with PET imaging is reliable. SEN of DC at the time of PET imaging was 79% overall and 78% for PVE, SEN of preoperatively performed FDG PET/CTs was 56% overall and 61% in PVE. Including positive FDG PET/CT scans as a major DC resulted in a gain of modified DC SEN in PVE cases by 16% (3 out of 4 patients with possible PVE were reclassified to definite PVE).

SEN values of DC range between 70 and 80% in literature,^11^ for PVE due to challenges in echocardiographic image acquisition and interpretation even lower values are observed.^10^ Hence, DC SEN values as detected in our study are well within the range of reported DC SEN.

In contrast, FDG PET/CT SEN in our current study was notably lower than SEN values in other studies. A meta-analysis of 13 studies involving 537 patients as published in 2017 found a pooled SEN of FDG PET/CT of 76.8% for all IE, respectively, of 80.5% for PVE.^12^ This discrepancy is somewhat surprising, especially since patients in our current study were all treated surgically suggesting a rather advanced stage of IE that should be more prone to pathologic FDG/PET CT scans. However, it needs to be mentioned that patients in our retrospective analysis represent an unselected real-world cohort of complexly diseased patients that ultimately required surgical therapy due to IE. Hence, indication for FDG PET/CT was not based on current ESC guidelines only. In fact, patients were mostly referred to the Department of Nuclear Medicine for the detection of an infectious focus in general. This is why usually recommended technical requirements such as the suppression of myocardial nuclide uptake or ECG-gated PET image acquisition were not routinely performed. These limitations in patient preparation and image acquisition may have likely contributed to the substantially lower sensitivity of FDG PET/CT in our study population that, however, still resulted in a relevant gain of diagnosing IE when using modified DC.

When comparing patients with true-positive vs false-negative scans in our study, median time since prosthesis valve implantation was significantly longer in the group with false-negative scans (median prosthesis age 6.5 years). In addition, proportion of patients with prostheses implanted longer than 1 year ago was significantly higher in patients with false-negative scans. Considering the lower PET/CT SEN in NVE^13^,^14^ this finding may be hypothesis generating in a way that valve prostheses may align their FDG PET/CT enhancement profile to NVE levels over time resulting in more false-negative scans. To the best of our knowledge this potential correlation has not been described before and should be taken into consideration when interpreting FDG PET/CT scans in patients with older valve prostheses.

Furthermore, we observed a trend toward less echocardiographically detected abscess formation (*P* = .053) and less female sex (*P* = .053) in patients with false-negative scans, yet without statistical significance. The former may suggest less inflammatory activity in patients with false-negative FDG PET/CT scans. However, in our study neither CRP nor leukocyte levels differed between both groups. This is in contrast to a study by Swart *et al* in which potential confounders of false-negative PET/CT scans in PVE patients were investigated and low inflammatory activity, namely low CRP levels at the time of PET imaging was described as a confounding factor resulting in false-negative scans.^10^ This discrepancy is hard to explain, but may be due to the fact that Swart *et al* have analyzed patients with less elevated overall inflammatory activity (CRP around 50-60 mg/L) who required surgical therapy in only half of the cases. Of note, patients in our study had *higher* CRP levels (ranging from 81-106 mg/L) and were all treated surgically, yet had a high proportion of false-negative FDG PET/CT scans. This may suggest a threshold from which elevated CRP levels in IE patients rather reflect the systemic inflammatory response as compared to the localized inflammatory activity within the IE affection site, and high CRP levels do not necessarily exclude the possibility of false-negative results.

Up to now, only one other study has investigated patients with preoperatively performed FDG PET/CTs and surgically managed IE.^15^ El-Dalati *et al* reported 12 true-positive FDG PET/CT scans in 12 patients with surgically proven IE (i.e., a SEN of 100%). Possible influencing factors like proportion of PVE, distribution of causative pathogens or surgical findings do not suggest such contradictory data. While a comprehensive explanation of these conflicting findings may be still limited to small sample size of both studies, further details of studied patients (e.g., time since prosthesis valve implantation or gender distribution) would be of interest in order to better understand possible confounders.

We only found few cases with NVE and cardiac device-related IE (CDRIE) in our study. In that scenario sensitivity of PET imaging was low (SEN NVE 33%, SEN CDRIE 50%) and addition to traditional DC did not result in a gain of SEN. For NVE low PET SEN has been reported so far with values ranging from 22% to 45%,^13^,^14^ in contrast Abikhzer et al. reported on notably higher SEN values 68%.^9^ Data to SEN in case of CDRIE are ranged from very low (16.3% in the ESC-EORP-EURO-ENDO study^14^) up to high values of > 85%.^5^,^7^ These conflicting data probably represent the heterogenic study situation in this highly complex disease.

According to the current ESC IE guidelines PET imaging should only be performed in suspected PVE with prostheses implanted more than 3 months ago to avoid false-positive scans as a result of postoperative inflammation. We included 4 patients with prostheses implanted *less* than 3 months ago and all of them had right positive FDG PET/CT scans. Despite lack of statistical significance this observation corroborates results from other studies^10^,^16^ questioning this 3 months safety period.

## Limitations

An important limitation of our study is the small number of patients in each group and the resulting lack of statistical power. Furthermore, due to the retrospective study design indication for FDG PET/CT was not based on current ESC guidelines only, but included also patients with definite IE diagnosis according to traditional DC which could lead to an important selection bias in this study.

Furthermore, our study may be influenced by a selection bias from identifying patients by the diagnosis and diagnostic procedures coded at the end of hospitalization for the statutory health insurance. From this reason, we expect a high prevalence of IE in our cohort. Because of our highly selected patient cohort determination of FDG PET/CT specificity as well as positive and negative predictive value is not viable. Unfortunately, data on pathological and microbiological evaluation of tissue samples were not available in all cases, which is relevant since definition of definite IE according to the current ESC IE guidelines does not involve sole confirmation of IE by the surgeon. ECG gating of PET images may increase detectability of small foci with elevated FDG uptake. In this retrospective study, only ungated PET images were analyzed as not all FDG PET/CTs were acquired with ECG gating, because most patients were referred to whole body PET/CT and not to dedicated cardiac PET imaging. Similarly, suppression of the myocardial nuclide uptake was not routinely performed, since the patients were referred to the Department of Nuclear Medicine for the detection of an infectious focus in general. These limitations in patient preparation and image acquisition may have likely contributed to the substantially lower sensitivity of FDG PET/CT in our study population.

## Conclusion

Our findings support the recommendation of current ESC IE guidelines for use of FDG PET/CT as complementary imaging to increase SEN of modified DC in PVE. However, we found evidence that increasing prosthesis age corresponds with lower SEN and therefore negative PET imaging should be interpreted with caution. Prospective trials are needed to better understand the value of FDG PET/CT in diagnosing IE.

## New Knowledge Gained

Our retrospective analysis that uses surgical assessment of definite IE diagnosis as reference standard gains knowledge with respect to the SEN of FDG PET/CT scans in a real-word cohort of unselected patients with surgically managed IE. We found a relevant gain of modified DC SEN (i.e., including FDG PET/CT scans and DC) in PVE patients only (3 out of 4 possible PVE patients could be reclassified to definite IE). PVE patients with false-negative FDG PET/CT results had significantly older valve prostheses, which should be considered when interpreting FDG PET/CT scans of IE patients.

## Supplementary Information

Below is the link to the electronic supplementary material.

Electronic supplementary material 1 (PPTX 13700 kb)

Electronic supplementary material 2 (M4A 928 kb)

## Abbreviations

CDRIE: Cardiac device-related infective endocarditis
DC: Duke criteria
ESC: European Society of Cardiology
FDG: ^18^F-fluorodeoxyglucose
ICD-10: International Classification of Diseases
IE: Infective endocarditis
NVE: Native valve endocarditis
PVE: Prosthetic valve endocarditis
SEN: Sensitivity
SUVmax: Maximal standardized uptake value
