# Supplementary material for: Diagnostic value of FDG PET/CT imaging in patients with surgically managed infective endocarditis: results of a retrospective analysis at a tertiary center
Source: J Nucl Cardiol. 2020 Dec 22;29(3):1191–204. doi: 10.1007/s12350-020-02457-x (PMC9162977; doi:10.1007/s12350-020-02457-x)
Supplement: Supplementary file 1 — Electronic supplementary material 1 (PPTX 13700 kb) [file 12350_2020_2457_MOESM1_ESM.pptx]

## Slide 1
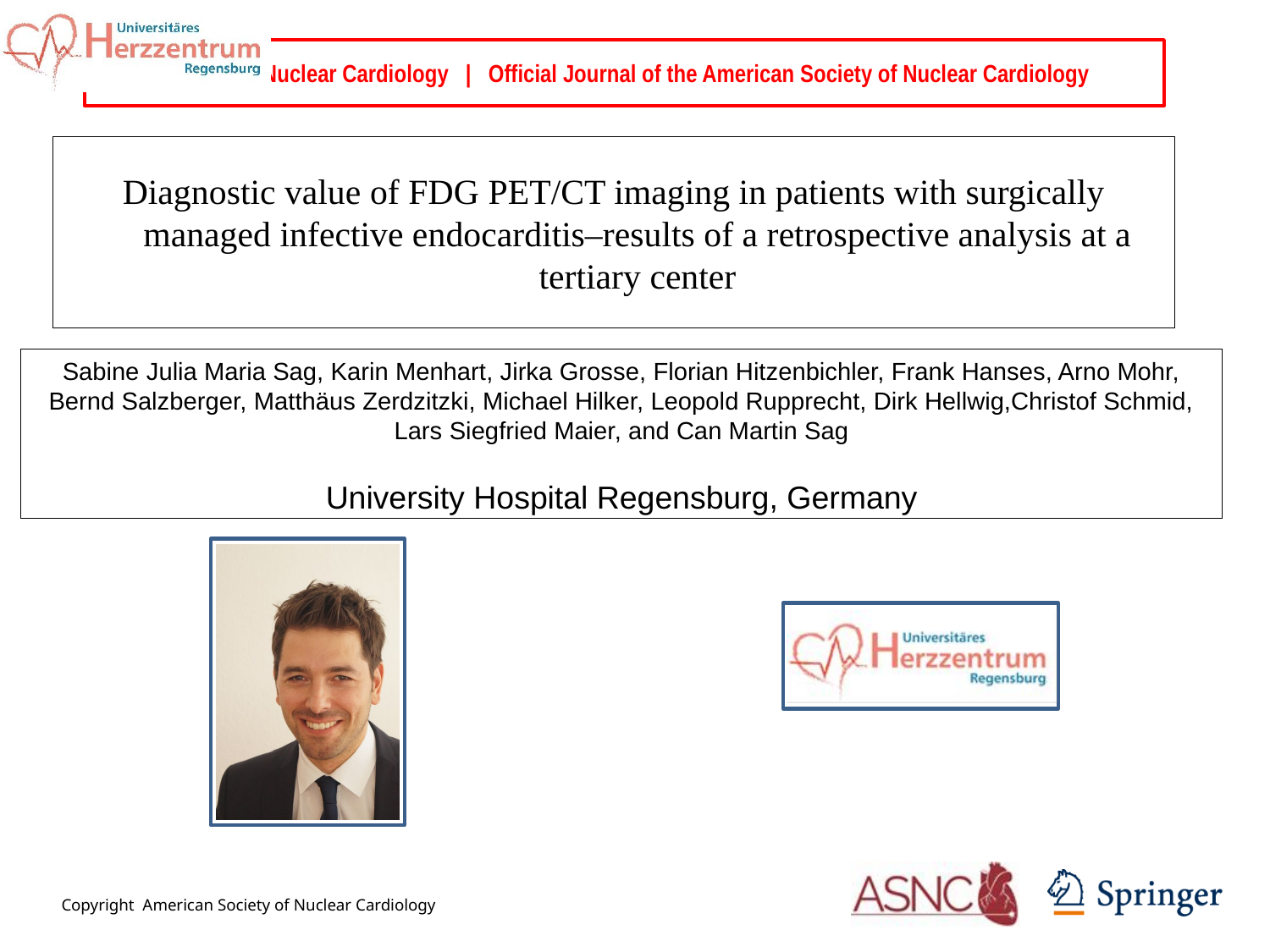

Journal of Nuclear Cardiology | Official Journal of the American Society of Nuclear Cardiology
# Diagnostic value of FDG PET/CT imaging in patients with surgically managed infective endocarditis–results of a retrospective analysis at a tertiary center
Sabine Julia Maria Sag, Karin Menhart, Jirka Grosse, Florian Hitzenbichler, Frank Hanses, Arno Mohr, Bernd Salzberger, Matthäus Zerdzitzki, Michael Hilker, Leopold Rupprecht, Dirk Hellwig,Christof Schmid, Lars Siegfried Maier, and Can Martin Sag
University Hospital Regensburg, Germany
Copyright American Society of Nuclear Cardiology

## Slide 2
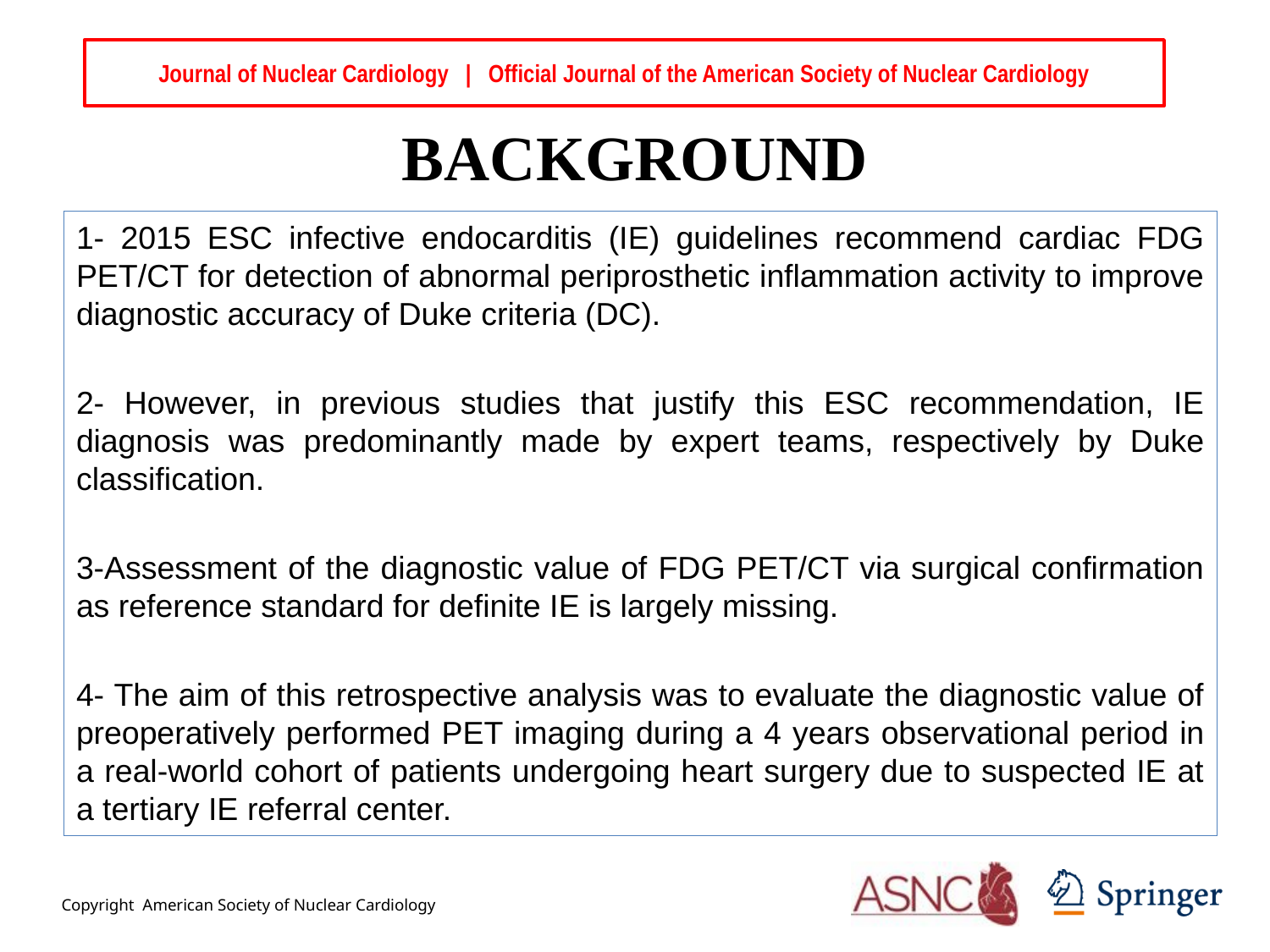

Journal of Nuclear Cardiology | Official Journal of the American Society of Nuclear Cardiology
# BACKGROUND
1- 2015 ESC infective endocarditis (IE) guidelines recommend cardiac FDG PET/CT for detection of abnormal periprosthetic inflammation activity to improve diagnostic accuracy of Duke criteria (DC).
2- However, in previous studies that justify this ESC recommendation, IE diagnosis was predominantly made by expert teams, respectively by Duke classification.
3-Assessment of the diagnostic value of FDG PET/CT via surgical confirmation as reference standard for definite IE is largely missing.
4- The aim of this retrospective analysis was to evaluate the diagnostic value of preoperatively performed PET imaging during a 4 years observational period in a real-world cohort of patients undergoing heart surgery due to suspected IE at a tertiary IE referral center.
Copyright American Society of Nuclear Cardiology

## Slide 3
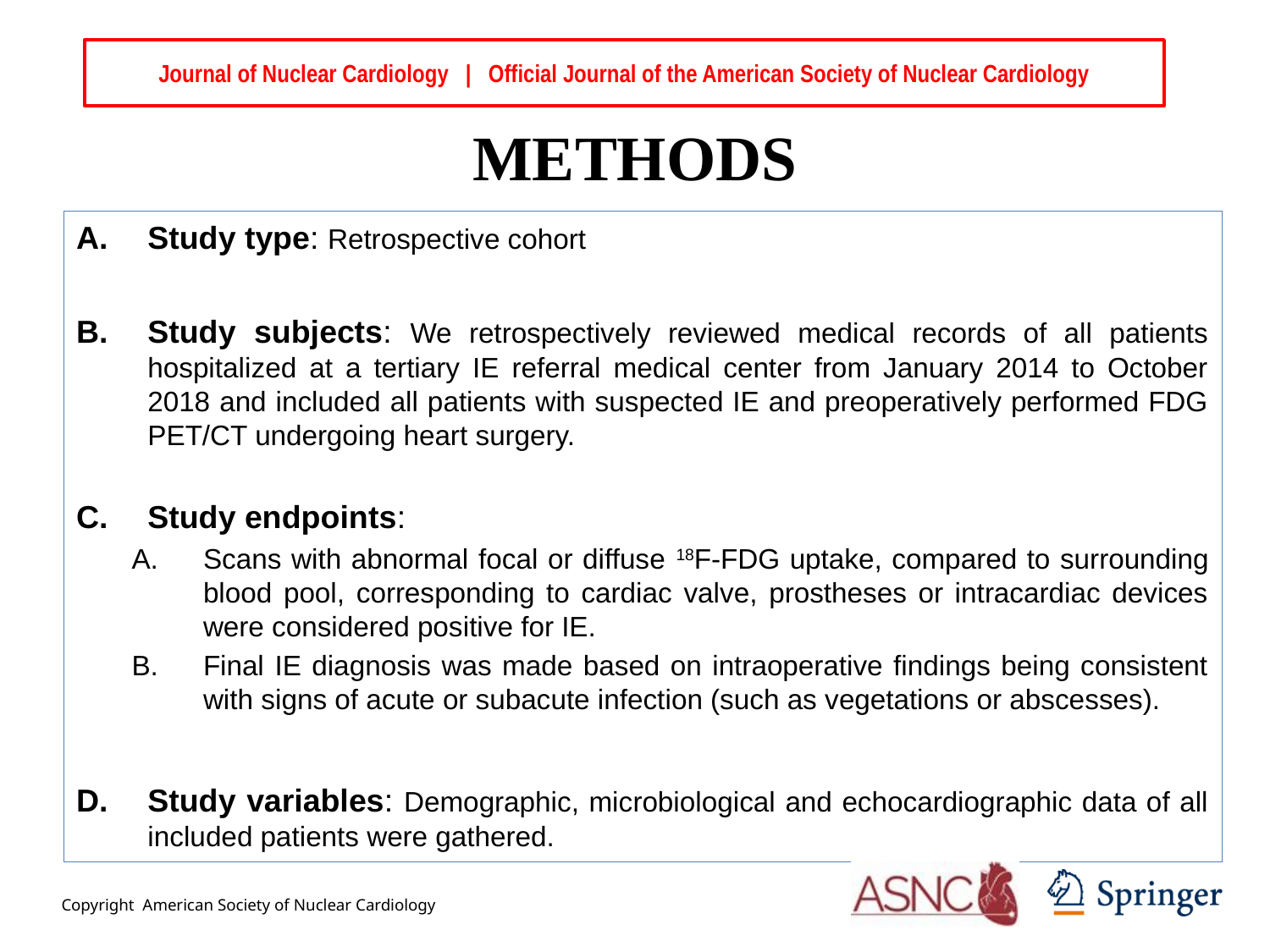

Journal of Nuclear Cardiology | Official Journal of the American Society of Nuclear Cardiology
# METHODS
Study type: Retrospective cohort
Study subjects: We retrospectively reviewed medical records of all patients hospitalized at a tertiary IE referral medical center from January 2014 to October 2018 and included all patients with suspected IE and preoperatively performed FDG PET/CT undergoing heart surgery.
Study endpoints:
Scans with abnormal focal or diffuse 18F-FDG uptake, compared to surrounding blood pool, corresponding to cardiac valve, prostheses or intracardiac devices were considered positive for IE.
Final IE diagnosis was made based on intraoperative findings being consistent with signs of acute or subacute infection (such as vegetations or abscesses).
Study variables: Demographic, microbiological and echocardiographic data of all included patients were gathered.
Copyright American Society of Nuclear Cardiology

## Slide 4
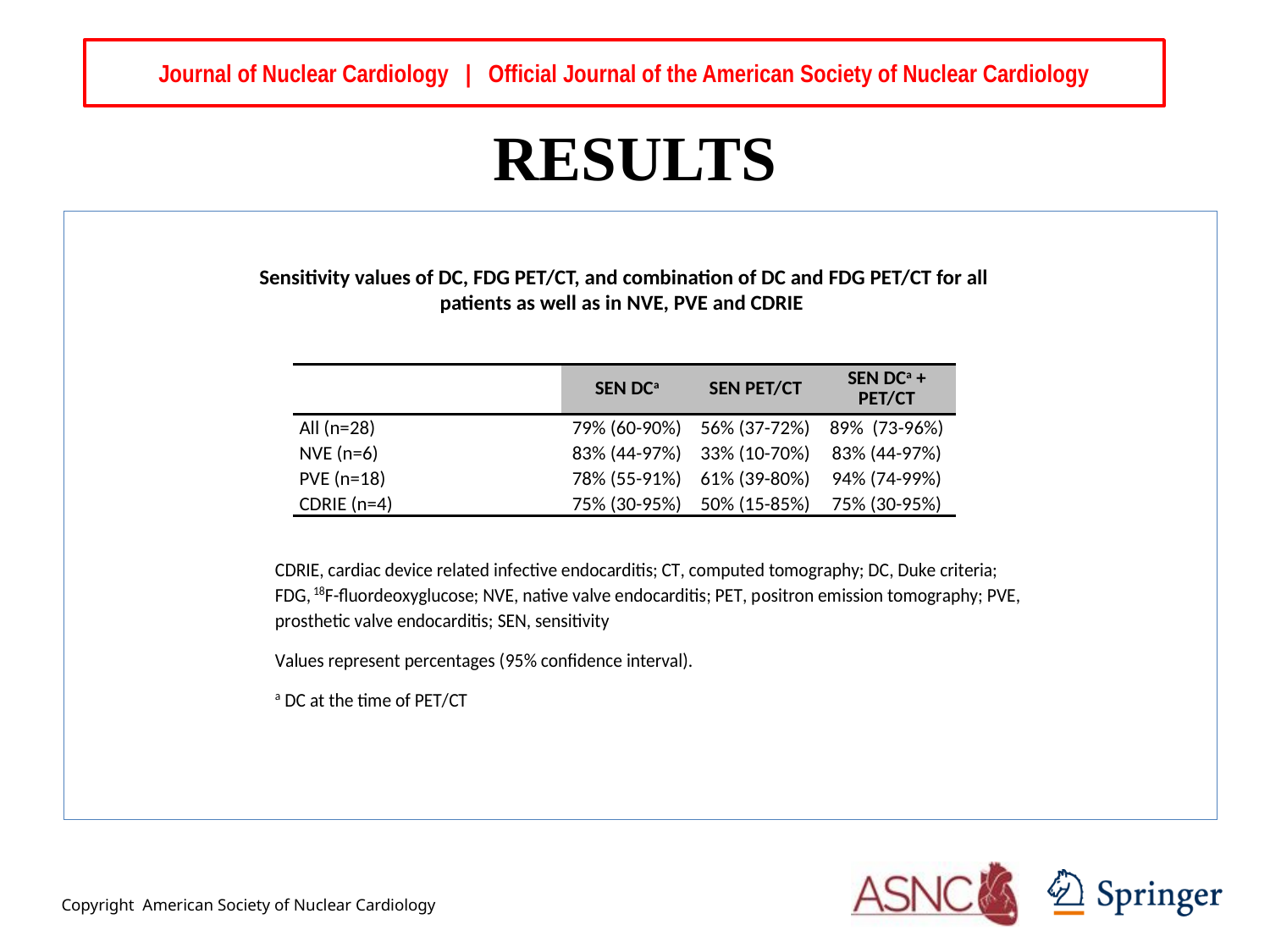

Journal of Nuclear Cardiology | Official Journal of the American Society of Nuclear Cardiology
# RESULTS
Sensitivity values of DC, FDG PET/CT, and combination of DC and FDG PET/CT for all patients as well as in NVE, PVE and CDRIE
| | SEN DCa | SEN PET/CT | SEN DCa + PET/CT |
| --- | --- | --- | --- |
| All (n=28) | 79% (60-90%) | 56% (37-72%) | 89% (73-96%) |
| NVE (n=6) | 83% (44-97%) | 33% (10-70%) | 83% (44-97%) |
| PVE (n=18) | 78% (55-91%) | 61% (39-80%) | 94% (74-99%) |
| CDRIE (n=4) | 75% (30-95%) | 50% (15-85%) | 75% (30-95%) |
Copyright American Society of Nuclear Cardiology

## Slide 5
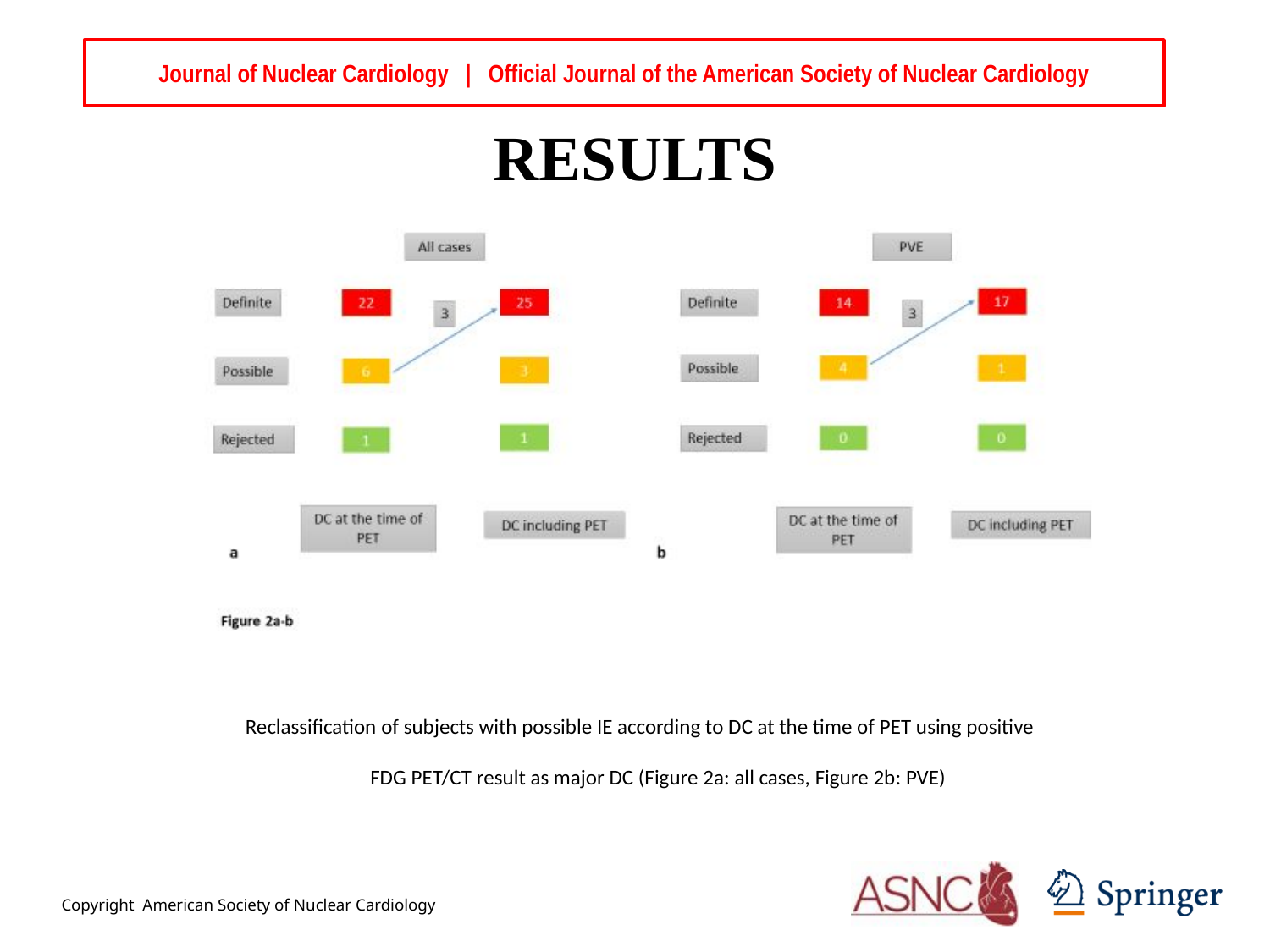

Journal of Nuclear Cardiology | Official Journal of the American Society of Nuclear Cardiology
# RESULTS
Reclassification of subjects with possible IE according to DC at the time of PET using positive FDG PET/CT result as major DC (Figure 2a: all cases, Figure 2b: PVE)
Copyright American Society of Nuclear Cardiology

## Slide 6
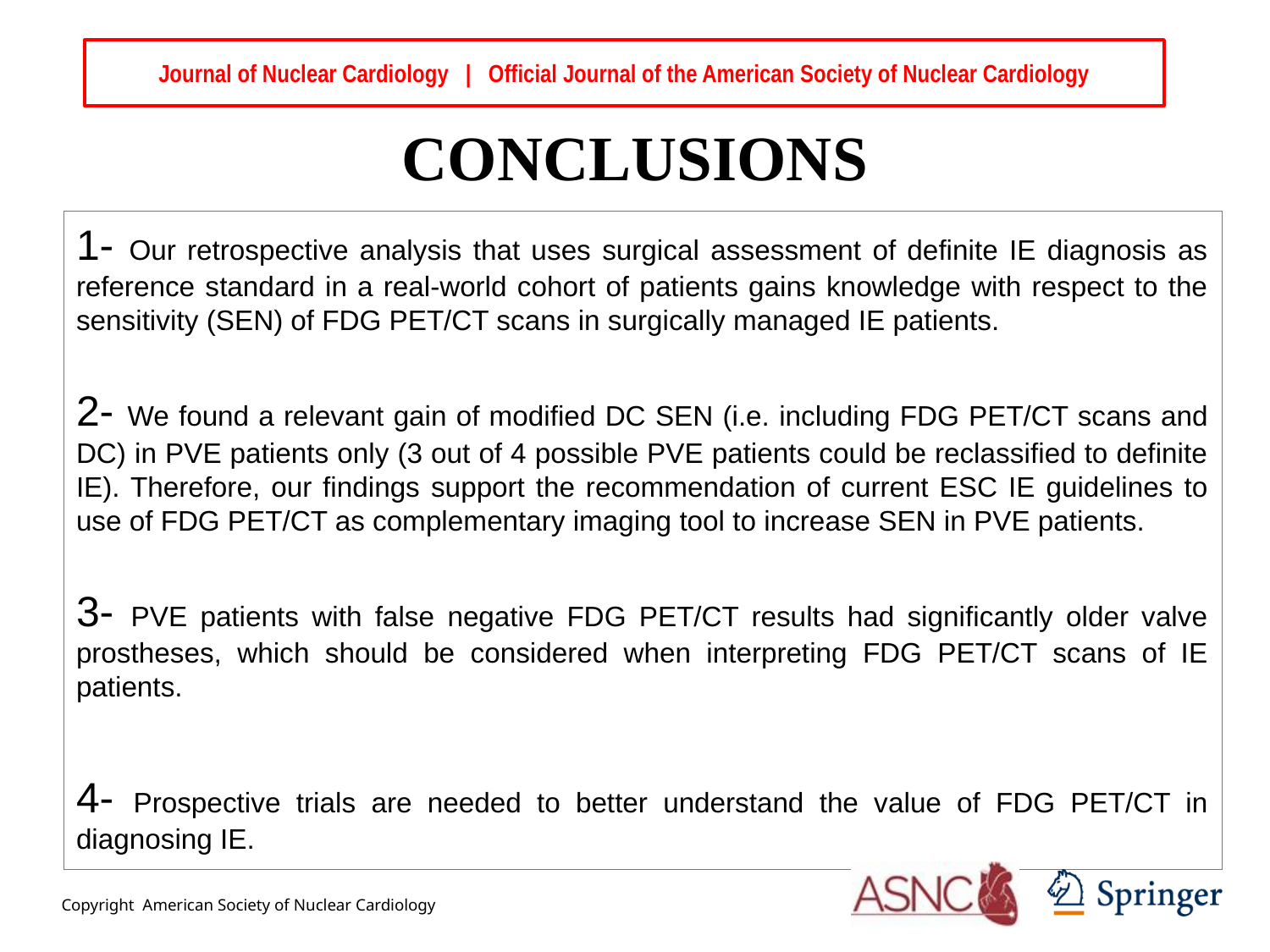

Journal of Nuclear Cardiology | Official Journal of the American Society of Nuclear Cardiology
# CONCLUSIONS
1- Our retrospective analysis that uses surgical assessment of definite IE diagnosis as reference standard in a real-world cohort of patients gains knowledge with respect to the sensitivity (SEN) of FDG PET/CT scans in surgically managed IE patients.
2- We found a relevant gain of modified DC SEN (i.e. including FDG PET/CT scans and DC) in PVE patients only (3 out of 4 possible PVE patients could be reclassified to definite IE). Therefore, our findings support the recommendation of current ESC IE guidelines to use of FDG PET/CT as complementary imaging tool to increase SEN in PVE patients.
3- PVE patients with false negative FDG PET/CT results had significantly older valve prostheses, which should be considered when interpreting FDG PET/CT scans of IE patients.
4- Prospective trials are needed to better understand the value of FDG PET/CT in diagnosing IE.
Copyright American Society of Nuclear Cardiology
